# Genome-wide association studies in horticultural crops: decoding genetic diversity for precision breeding

**DOI:** 10.1093/hr/uhag059

**Published:** 2026-02-27

**Authors:** Dandan Lou, Yuyao Zhang, Pengchuan Wu, Hui Xiao, Fei Guo, Xingtan Zhang, Pu Wang, Weilong Kong

**Affiliations:** College of Horticulture and Forestry Sciences, Hubei Hongshan Laboratory, National Key Laboratory for Germplasm Innovation and Utilization of Horticultural Crops, Key Laboratory of Horticultural Plant Biology, Ministry of Education, Hubei Engineering Technology Research Center for Forestry Information, Huazhong Agricultural University, Wuhan 430070, China; Yuelushan Laboratory, Changsha 410128, China; Shenzhen Branch, Guangdong Laboratory for Lingnan Modern Agriculture, Genome Analysis Laboratory of the Ministry of Agriculture, Agricultural Genomics Institute at Shenzhen, Chinese Academy of Agricultural Sciences, Shenzhen 518120, China; College of Horticulture and Forestry Sciences, Hubei Hongshan Laboratory, National Key Laboratory for Germplasm Innovation and Utilization of Horticultural Crops, Key Laboratory of Horticultural Plant Biology, Ministry of Education, Hubei Engineering Technology Research Center for Forestry Information, Huazhong Agricultural University, Wuhan 430070, China; College of Horticulture and Forestry Sciences, Hubei Hongshan Laboratory, National Key Laboratory for Germplasm Innovation and Utilization of Horticultural Crops, Key Laboratory of Horticultural Plant Biology, Ministry of Education, Hubei Engineering Technology Research Center for Forestry Information, Huazhong Agricultural University, Wuhan 430070, China; College of Horticulture and Forestry Sciences, Hubei Hongshan Laboratory, National Key Laboratory for Germplasm Innovation and Utilization of Horticultural Crops, Key Laboratory of Horticultural Plant Biology, Ministry of Education, Hubei Engineering Technology Research Center for Forestry Information, Huazhong Agricultural University, Wuhan 430070, China; College of Horticulture and Forestry Sciences, Hubei Hongshan Laboratory, National Key Laboratory for Germplasm Innovation and Utilization of Horticultural Crops, Key Laboratory of Horticultural Plant Biology, Ministry of Education, Hubei Engineering Technology Research Center for Forestry Information, Huazhong Agricultural University, Wuhan 430070, China; Shenzhen Branch, Guangdong Laboratory for Lingnan Modern Agriculture, Genome Analysis Laboratory of the Ministry of Agriculture, Agricultural Genomics Institute at Shenzhen, Chinese Academy of Agricultural Sciences, Shenzhen 518120, China; College of Horticulture and Forestry Sciences, Hubei Hongshan Laboratory, National Key Laboratory for Germplasm Innovation and Utilization of Horticultural Crops, Key Laboratory of Horticultural Plant Biology, Ministry of Education, Hubei Engineering Technology Research Center for Forestry Information, Huazhong Agricultural University, Wuhan 430070, China; College of Horticulture and Forestry Sciences, Hubei Hongshan Laboratory, National Key Laboratory for Germplasm Innovation and Utilization of Horticultural Crops, Key Laboratory of Horticultural Plant Biology, Ministry of Education, Hubei Engineering Technology Research Center for Forestry Information, Huazhong Agricultural University, Wuhan 430070, China; Yuelushan Laboratory, Changsha 410128, China; Shenzhen Branch, Guangdong Laboratory for Lingnan Modern Agriculture, Genome Analysis Laboratory of the Ministry of Agriculture, Agricultural Genomics Institute at Shenzhen, Chinese Academy of Agricultural Sciences, Shenzhen 518120, China

## Abstract

Horticultural crops, including fruits, vegetables, ornamental plants, and tea plants, are vital for economic and nutritional sustainability, yet their cultivation is severely hampered by abiotic stresses such as heat, cold, and salinity. The advent of the grapevine genome in 2007 initiated the genomic era for horticultural species. This milestone facilitated the use of genome-wide association studies (GWAS) to decode the complex phenotypic diversity of these crops. Unlike traditional methods, GWAS utilizes natural genetic diversity to identify quantitative trait loci linked to key traits, offering a high-resolution approach for dissecting traits such as stress resistance, quality, and yield. This review highlights the innovative workflows and technical advancements in GWAS applications for horticultural crops, covering aspects including population design, high-throughput phenotyping, sophisticated statistical modeling, and their applications in horticultural plants. Notably, the integration of multi-omics approaches has enhanced our understanding of the genetic mechanisms underlying critical horticultural traits. Future directions aim at harnessing technological innovations, cross-omics synthesis, and precision breeding strategies to optimize trait selection and expedite the development of resilient cultivars. Consequently, GWAS serves as a crucial bridge linking genomic variation to practical applications in horticultural improvement, enabling a paradigm shift toward predictive breeding and sustainable agricultural practices.

## Introduction

Horticultural crops, encompassing diverse fruits, vegetables, ornamental plants, and tea plants, hold substantial economic and societal importance. According to the Food and Agriculture Organization (FAO, http://faostat.fao.org/), global production of fruits and vegetables in 2023 reached 951.9 million tonnes and 1186.7 million tonnes, respectively. The combined market value of this produce exceeded USD 970.66 billion. Beyond economic contributions, horticultural crops constitute vital sources of essential vitamins, minerals, and dietary fiber in human diets [1, 2] and possess ecological, ornamental (e.g. flowers), and medicinal value (e.g. Asteraceae plants [3]). However, the cultivation of horticultural crops faces severe constraints from abiotic stresses, including heat, cold, waterlogging, and salinity [4, 5]. These stresses impede growth, reduce yield, and limit geographical adaptability. Understanding the genetic basis underlying plant responses to these stresses and the natural variation in stress tolerance among different varieties is therefore crucial for developing resilient crops.

The publication of the grapevine (*Vitis vinifera*) genome in 2007 [6] initiated the genomics era for horticultural crops, enabling systematic dissection of their extensive phenotypic diversity. Unlike major cereals, horticultural crops exhibit exceptional phenotypic variation, spanning complex traits including flower morphology, color, leaf shape, fruit flavor, and flowering time, and critically, responses to environmental stresses [7, 8]. This diversity arises from prolonged domestication and manual selection [9], resulting in genomic heterogeneity, a feature seen in polyploid species like strawberry [10] and tree peony that complicates genetic analysis.

Genome-wide association studies (GWAS) utilize linkage disequilibrium to associate multiple genomic variants such as single nucleotide polymorphisms (SNPs), structural variations (SVs), and insertion/deletion polymorphisms (InDels) with phenotypic variation in natural populations, thereby identifying trait-governing QTLs. Unlike traditional linkage analysis reliant on biparental crosses, GWAS utilizes natural genetic diversity, bypassing artificial population construction [11]. This is a critical advantage for perennial horticultural crops with complex genetic backgrounds. Compared to conventional linkage mapping, GWAS provides three advantages: (i) GWAS permits direct utilization of existing natural populations, eliminating the requirement for specialized mapping populations and thus offering a time- and resource-efficient strategy [12]; (ii) high-resolution mapping capability based on LD enabling fine-mapping to single-gene precision [13]; and (iii) efficacy in deconstructing polygenic regulatory networks. In horticulture, GWAS has successfully identified genes associated with stress resistance, quality, and yield traits [14, 15], delivering targets for marker-assisted selection and accelerating genetic improvement significantly.

This review integrates technical workflows, innovative strategies, and trait dissection advances in horticultural GWAS research. We herein emphasized integrative multi-omics applications and project future advancements in precision breeding, establishing a theoretical framework for horticultural genetic enhancement.

## Precision-engineered GWAS: tech-driven workflow innovations in horticultural crops

**Figure 1:**
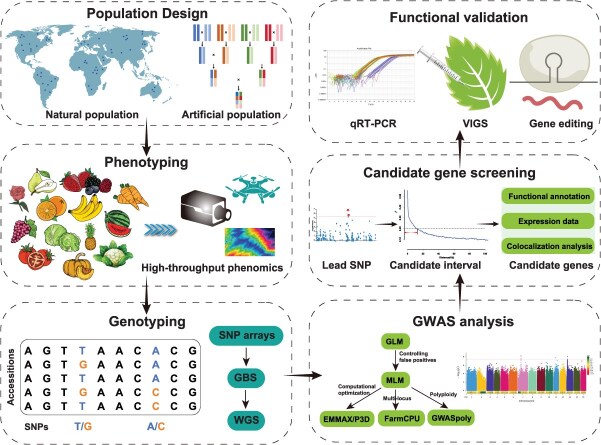
Integrated precision GWAS workflow for horticultural crop improvement.

As a pivotal technique for decoding the genetic architecture of complex traits in horticultural crops, GWAS efficacy depends on a rigorous scientific workflow. Synergistic innovations across population design, high-throughput phenotyping, genotyping technologies, and statistical modeling [16] propel transformative advances in functional gene discovery and molecular breeding (Fig. 1).

### Population design and optimization

GWAS implementation begins with scientifically optimized population design. Natural diversity panels represent a foundational strategy; large-scale sampling of 200–2000 accessions typically captures >97% of common allelic variants (MAF > 0.05) [12]. However, population structure resulting from domestication bottlenecks imposes limitations on mapping resolution (typically 10–100 kb). This genome-wide LD enhancement, induced by population bottlenecks, dilutes association signals across broad genomic regions and impedes precise identification of causal variants. Multiparent advanced generation intercross (MAGIC) populations overcome this limitation by enabling effective reduction of LD through prolonged random mating. In lettuce (*Lactuca sativa*), a MAGIC population constructed through cyclic crosses of 16 founders reduced population structure interference and achieved 1–5 kb fine-mapping resolution [17]. In addition, population structure stratification can confound genuine genotype–phenotype associations, generating substantial false-positive signals and masking authentic causal variants. Incorporating population structure matrices (e.g. PCA or kinship) as covariates in statistical models effectively controls these spurious associations [18]. For pronounced stratification, meta-GWAS with stratified analysis detects subpopulation-specific alleles while correcting false positives. In rice (*Oryza sativa*), stratified analysis of 7765 accessions across six genetic groups identified 156 QTLs—including 116 novel loci exclusively detected through meta-analysis [19].

### Phenomic analysis

Precise phenotyping remains challenging due to the complexity and polymorphism of horticultural traits. Traditional manual methods exhibit low throughput and subjectivity. Recent breakthroughs in high-throughput phenomics significantly enhance GWAS capabilities. For example, hyperspectral imaging capturing the 401 -998-nm spectral range enables quantification of physiological indices, including chlorophyll and anthocyanin content in lettuce. This approach precisely mapped nine QTLs, with four colocalizing with known leaf-color genes [17]. Unmanned aerial system-based RGB imaging platforms have also proven effective for high-throughput phenotyping of growth dynamics like canopy coverage and volume in spinach (*Spinacia oleracea*). This approach revealed 99 loci significantly associated with growth traits [20]. Tomato (*Solanum lycopersicum*) studies further demonstrate high-throughput phenotyping efficacy. Rodriguez *et al.* [21] quantified over 40 fruit morphological traits digitally, identifying 536 association loci and highlighting landraces’ utility for complex trait genetics.

Key horticultural traits (yield, quality, stress resilience) exhibit substantial plasticity across years and environments in multilocation trials. This environmental plasticity, driven by genotype-by-environment (G × E) interactions, necessitates comprehensive phenotyping across diverse environments to accurately capture genetic potential and trait stability. While high-throughput phenomics enables scalable data capture, environmental noise complicates signal extraction. Best Linear Unbiased Prediction (BLUP) is a statistical method that accounts for environmental and genetic noise by integrating multiyear and multilocation data. It helps extract stable genetic signals from noisy phenotypic datasets. In sesame (*Sesamum indicum* L.), BLUP analysis of seed coat color across 12 environments identified 119 significant SNPs, with all environmentally stable SNPs (>6 environments) detected using BLUP values [22]. This approach minimizes noise and enhances genetic signal detection for downstream analyses.

### Genotyping

Declining costs and improved precision of genotyping have significantly propelled GWAS applications in horticulture. The field has shifted paradigms from SNP arrays (e.g. strawberry’s 50k FanaSNP array [23]) to sequencing-dominated approaches. At present, genotyping-by-sequencing and whole-genome resequencing (WGS) are mainstream [15, 24]. Capitalizing on its comprehensive genome coverage, WGS represents the gold standard for resolving complex genetic variations, enabling efficient detection of SNPs, copy number variations (CNVs) [25], and even SVs [26]. A significant limitation of conventional SNP-based GWAS emerges in WGS analyses: short-read mapping strategies against a single reference genome frequently fail to accurately resolve complex SVs. This is particularly true for SVs that are absent from the reference assembly or are located within repetitive or structurally dynamic genomic regions. This reference bias consequently obscures associations for these genomic features. Graph-genome technology addresses this limitation by integrating pan-genomic diversity into reference structures, enabling improved short-read alignment and sensitive SV detection across diverse germplasm. For instance, graph-pangenome-based GWAS technology identified a 347-bp deletion SV at 65.2 Mb on chromosome 10, significantly affecting geranylacetone content (*P* = 7.91 × 10^−9^)—undetectable via SNP-based GWAS [27].

### GWAS statistical models

The core of GWAS relies on statistical models associating genotypes with phenotypes (Table 1). Model selection is crucial and depends on population structure, trait genetic architecture, and data type. Initially, the general linear model (GLM) was widely adopted for its computational efficiency and simplicity. However, GLM is prone to false positives due to unaccounted population stratification and kinship [28]. Mixed linear model (MLM) addresses this by incorporating kinship matrices as random effects, establishing MLM as a standard framework [29, 30]. To overcome the computational burden of MLMs, optimized algorithms (e.g. EMMA, P3D/EMMAX, FaST-LMM/GEMMA) have been developed, significantly enhancing feasibility for large-scale studies [31–35]. For complex polygenic traits, where MLM may overcorrect and reduce statistical power, advanced methodologies offer improved sensitivity. Multilocus models (e.g. FarmCPU, BLINK) enhance detection power for small-effect loci [36, 37]. Optimized MLM variants (e.g. CMLM/ECMLM) and SNP selection models (e.g. FaST-LMM-Select, SUPER) further improve efficiency and power [32, 38–40]. Alternatively, Bayesian approaches (e.g. BayesA/B) jointly estimate genome-wide effects using prior distributions, circumventing multiple testing issues, and are suited for traits with specific genetic architectures [41].

**Table 1:** GWAS models in horticulture: strengths, weaknesses, and implementation.

**Model category**	**Representative method(s)**	**Relevant software/tool**	**Key advantages**	**Limitations**	**Application in horticultural crops**
Basic Single-Locus Models	GLM	TASSEL, GAPIT, GEMMA, rMVP	Computationally efficient and straightforward to implement.	Susceptible to confounding effects from population structure and kinship, leading to elevated false positives.	Rarely used alone; typically serves as a baseline for comparison or preliminary screening. Performance is often compromised by population structure in horticultural crops.
	MLM	GAPIT, TASSEL, GEMMA, GCTA, rMVP	Effectively controls population structure and kinship via a kinship matrix (random effect), significantly reducing false positives	Computationally intensive; reduced power to detect small-effect polygenic loci, increasing false negatives.	Most widely applied; foundational model for horticultural GWAS. Suitable for crops with complex demographic histories (e.g. domestication, breeding). May miss subtle effects in highly polygenic traits.
Computational Efficiency-Optimized Models	EMMAX/P3D	EMMAX, GAPIT	Dramatically accelerates analysis by preestimating variance components, avoiding per-SNP recalculation; ideal for large cohorts.	Fixed variance components may reduce precision and flexibility under complex genetic architectures.	Routinely used for large-scale horticultural populations (e.g. core collections, natural populations) to expedite workflows.
	GEMMA	GEMMA, GAPIT	High computational efficiency via reformulated likelihood functions and matrix decomposition; supports parallelization.	Memory intensive	Applied for rapid analysis of moderate-to-large horticultural cohorts.
Statistical Power-Enhanced Models	FarmCPU	GAPIT, rMVP	Balances false negatives and false positives via iterative covariate screening; superior power for small-effect polygenic loci.	Parameter tuning (e.g. iterations, covariate selection) is experience dependent; moderate computational overhead.	Increasingly adopted for complex quantitative traits (e.g. stress resilience, quality). Excels in horticultural traits governed by numerous small-effect genes, uncovering more candidate loci.
	BLINK	GAPIT, BLINK	Superior power in detecting association signals with the ability to analyze massive datasets.	Less flexible for traits with extreme polygenicity; requires careful parameter calibration.	Gaining traction for dissecting complex horticultural traits with polygenic architecture, especially when high statistical power is required.
	CMLM/ECMLM	GAPIT, TASSEL	Maintains accuracy while accelerating computation by compressing (clustering) kinship matrices to reduce random-effect parameters.	Overcompression may mask within-subpopulation genetic variation; sensitive to fine-scale population structure.	Used for horticultural populations with pronounced substructure (e.g. distinct geographic origins, varietal groups) as a power-speed trade-off.
	FaST-LMM-Select	FaST-LMM, GAPIT	Enhances detection of polygenic effects by constructing trait-informed kinship matrices from selected SNP subsets.	SNP subset selection may introduce bias, omitting weak-effect loci or variants unlinked to selected SNPs.	Suitable for studies prioritizing trait-relevant genetic backgrounds, boosting detection in target genomic regions or locus types.
Bayesian Models	BayesA/B	BGLR	Circumvents multiple testing correction via joint estimation of genome-wide effects; well suited for large-effect loci or highly polygenic traits.	Extremely computationally intensive; sensitive to prior distribution specifications; complex implementation and interpretation.	Limited adoption in horticulture due to computational demands.
Polyploid-Specific Models	GWASpoly	GWASpoly	Supports polyploid genotype encoding (e.g. AAAA/AAAa), models dosage effects (additive, dominant); integrates DAPC for improved structure correction.	Requires high-quality polyploid genotyping (precise dosage calls) and dense markers; sensitive to genotyping errors.	Essential for key polyploid horticultural crops (e.g. potato, strawberry, peony). Accurately associates gene dosage with phenotypic variation, enabling robust genetic dissection of complex traits.
	KMERIA	KMERIA	Reference-genome-free; detects diverse variants (SVs, PAVs) via k-mer abundance association; natively models polyploid dosage effects.	Substantial storage/computational demands for k-mer databases; complex association testing; requires additional steps for variant interpretation.	Emerging method; not yet applied but especially adapted for polyploid horticultural crops lacking reference genomes or rich in SVs/PAVs (e.g. strawberry, ornamentals). Uncovers variation missed by SNP-based methods.
Deep Learning Models	DeepGWAS	DeepGWAS	Capture nonlinear and high-order genetic interactions; improved detection of small-effect or context-dependent loci	Limited interpretability; higher computational cost; require large training datasets	Trait prediction and candidate gene prioritization in complex traits, particularly in polyploid crops such as potato and strawberry

In addition to conventional linear and Bayesian models, deep learning (DL)-based approaches have recently emerged as an extension of GWAS analytical frameworks. By learning nonlinear and high-dimensional feature representations, DL models show particular potential for dissecting complex horticultural traits involving epistasis, allelic dosage effects, and genotype-by-environment interactions [42]. Representative deep learning frameworks such as DeepGWAS enhance association signals for loci with small or context-dependent effects [43]. Recent studies further demonstrate that integrating deep learning architectures with k-mer-based or multivariant genomic representations improves trait prediction and candidate gene prioritization, particularly in polyploid crops [44]. Notably, DL-based GWAS is generally applied in conjunction with, rather than as a replacement for, classical statistical models. Integrating DL-derived predictions or feature importance with conventional GWAS results offers a pragmatic strategy to improve locus discovery and biological interpretation in horticultural genomics.

This integrative modeling strategy is particularly relevant for horticultural crops with complex genomic architectures. Many important horticultural crops including potato (*Solanum tuberosum*), strawberry (*Fragaria × ananassa*), and peony (*Paeonia suffruticosa*) frequently exhibit autopolyploid or allopolyploid genomes. These polyploid complexities challenge conventional diploid-oriented GWAS models. A core bottleneck in polyploid genomics is accurate dosage calling, which involves determining the precise copy number of alleles (e.g. AAAA vs AAAa in a tetraploid). This task remains challenging due to the presence of multiple homologous copies in polyploids, resulting in complex allele dosage patterns. These patterns are difficult to resolve using short-read sequencing, particularly in regions with high heterozygosity or structural complexity. Inaccurate dosage calls can obscure true genotype–phenotype associations and reduce mapping resolution. Beyond dosage estimation, allopolyploid species introduce an additional layer of complexity arising from the presence of multiple subgenomes derived from distinct progenitor species. In such genomes, homoeologous loci may exhibit subgenome-specific sequence divergence, expression dominance, and asymmetric phenotypic contributions. Consequently, accurate detection of subgenome-specific QTLs represents a critical yet underexplored challenge in horticultural GWAS, particularly in crops such as cultivated strawberry, where key agronomic and quality traits are often controlled by loci residing on specific subgenomes. Specialized tools like GWASpoly are designed for polyploids, employing genotype encodings that discriminate allelic dosage and model dosage effects (additive, dominant). By integrating dosage information directly into association tests, GWASpoly reduces common genotyping errors in polyploids and improves the detection of gene dosage-dependent causal variants. This allows accurate association of gene dosage with phenotypic variation [45]. However, resolving the subgenome origin of associated signals still relies heavily on high-quality, subgenome-resolved reference genomes. Furthermore, k-mer-based GWAS offers a powerful, reference-free solution for polyploid species. It detects diverse variation types, including SVs and presence/absence polymorphisms, while naturally incorporating allelic dosage effects. Since k-mers are counted directly from sequencing reads without reference alignment, their abundance directly reflects genomic copy number. This property makes k-mer-based approaches particularly promising for capturing subgenome-specific variation in highly heterozygous allopolyploid genomes, even without a high-quality reference genome or precise variant calling [46, 47].

In horticultural genomics, GLM/MLM variants remain predominant, while multilocus models are increasingly adopted for complex traits. Bayesian approaches see limited adoption due to computational intensity. Model selection must integrate population parameters, trait genetic architecture, and data quality. Its validity is typically assessed using Q–Q plots. Increasingly adopted in current research, multimodel GWAS frameworks integrate complementary statistical approaches to overcome single-model limitations. This strategy provides a comprehensive genetic dissection of complex traits, identifies concordant loci for validation, and accelerates molecular breeding pipelines.

### Candidate gene screening

After identifying significant association signals from GWAS, the next critical step is to pinpoint the most likely causal gene(s) within the candidate genomic regions. This bridges statistical discovery with functional validation. Due to linkage disequilibrium, a significantly associated SNP frequently does not represent the causal variant but is instead a marker in LD with it. Consequently, defining the precise physical interval harboring the association signal is paramount. A standard approach defines the candidate interval using the population’s LD decay distance, extending from the lead SNP until the linkage disequilibrium coefficient (*r*^2^) falls below a predefined threshold [19]. In populations with high-resolution fine-mapping capacity, such as MAGIC populations, this interval may be refined to within several kilobases. Functional annotation (e.g. GO and KEGG pathway enrichment analyses) of genes within the candidate interval to screen those involved in biological processes related to the target trait represents a common strategy. Integration of expression data (e.g. from RNA sequencing) from trait-relevant tissues, developmental stages, or experimental conditions can significantly strengthen candidate gene plausibility [48]. Furthermore, colocalization analysis evaluates the genetic overlap between association signals for distinct traits (e.g. a major QTL and an expression QTL) or for the same trait across different environments within a shared genomic region. This approach offers a robust strategy for identifying putative causal genes underlying the observed associations.

### Functional validation

Functional validation confirms GWAS-identified gene-trait causality. Establishing definitive causality typically requires multilevel validation spanning transcriptional, protein, and functional tiers (Fig. 1). At the transcriptional level, techniques including RNA-seq, qRT-PCR, and semiquantitative PCR quantify expression differences in individuals with contrasting phenotypes, directly linking transcript abundance to trait variation. As demonstrated in tomato, qRT-PCR confirmed cold-induced upregulation of the GWAS-identified *SlBBX31*, with significantly higher expression in cold-tolerant genotypes than cold-sensitive counterparts [2]. At the protein level, methods including immunoprecipitation (Co-IP), Western blotting, and protein quantification assays validate whether genetic variants alter protein abundance, modification, or interaction—critical for traits governed by post-transcriptional regulation. However, expression or protein correlation alone is insufficient. Definitive functional validation at the organism level is essential to directly evaluate the regulatory impact of a candidate gene on target traits. For instance, overexpression assays in apple have validated the functions of mGWAS-identified genes. Transient overexpression of *MdNAP* significantly elevated SA accumulation. In contrast, plants overexpressing *MdABCG25* showed significantly increased ABA levels relative to controls. Together, these results demonstrate that both genes suppress cell expansion and induce small fruit phenotypes through hormone pathways [49]. Loss-of-function approaches, such as gene silencing or knockout, reveal phenotypic deficiencies upon suppression, offering reverse-genetic validation of causality. In pear (*Pyrus pyrifolia*) stone cell formation research, transient suppression of the GWAS-identified key gene *PbrSTONE* using virus-induced gene silencing (VIGS) technology significantly reduced stone cell content and lignin deposition in the fruit [50].

### Integrative and derived analytical frameworks

While GWAS is powerful for identifying genotype–phenotype associations, several derived or complementary analytical approaches have been developed to enhance causal inference and deepen mechanistic insights. These approaches include transcriptome-wide association studies (TWAS), expression quantitative trait locus (eQTL) mapping, meta-GWAS, metabolome GWAS (mGWAS), and TE-GWAS, among others. These methods extend GWAS beyond simple marker-trait associations, enabling deeper insights into regulatory mechanisms, polygenic architecture, and practical breeding applications.

TWAS and eQTL mapping bridge genomic variation to gene expression. TWAS integrates gene expression data with genetic variation to identify genes whose expression levels are associated with phenotypic variation. Unlike GWAS, which tests direct genotype–phenotype associations, TWAS uses predicted gene expression as an intermediate trait, thereby identifying genes whose regulation may drive phenotypic differences. Conversely, eQTL mapping identifies genomic regions associated with variation in gene expression levels. When combined with GWAS, eQTL analysis helps determine whether trait-associated variants exert their effects through modulation of gene expression. In pepper (*Capsicum annuum*), integrated TWAS and eQTL mapping within a recombinant inbred line population identified 12 core QTLs for fruit color, width, length, and weight. It also highlighted key regulatory hotspots that coordinate transcriptional and translational control during fruit development [51].

Meta-GWAS combines association results from multiple independent studies to increase statistical power, improve detection of small-effect loci, and enable cross-population validation. By aggregating data across diverse genetic backgrounds and environments, meta-GWAS can identify consistent loci and reveal population-specific associations. In a multi-environment study of peach (*Prunus persica*) and apricot (*Prunus armeniaca*), meta-GWAS integrating data from multiple sites and years identified 60 high-confidence QTLs for resistance to several biotic stresses. Among these, a stable QTL on chromosome 4 was consistently associated with leaf curl resistance in peach, demonstrating the utility of meta-GWAS in uncovering robust, environment-shared loci [14].

Bulked segregant analysis sequencing (BSA-seq) offers a rapid, cost-effective method for mapping qualitative or major-effect quantitative trait loci. It works by comparing allele frequencies in pooled DNA from extreme phenotypic bulks of a segregating population. Unlike GWAS, which harnesses natural diversity, BSA-seq typically uses biparental or multiparental populations, providing high power for initial QTL localization with minimal population structure confounding. In tomato, applying BSA-seq to an F₂ population quickly mapped a major malate QTL (TFM6) to a 9.6 Mb interval. Subsequent work fine-mapped and validated it as *Sl-ALMT9*, showcasing BSA-seq’s effectiveness for initial QTL discovery [52].

In summary, the integration of GWAS with complementary approaches such as TWAS, eQTL mapping, meta-GWAS, and BSA-seq provides a multidimensional framework for moving from genetic association to causal mechanism and functional insight. These methods are increasingly being adopted in horticultural genomics to decipher complex traits, prioritize candidate genes, and accelerate precision breeding.

## Dissecting genetic mechanisms of key horticultural traits

Building upon the rigorous technical workflows and innovative strategies outlined in the preceding section, GWAS has been extensively applied to decode the genetic architecture of key horticultural traits. Capitalizing on its capacity to efficiently dissect complex traits, GWAS has substantially advanced understanding of key horticultural traits. These breakthroughs center on core agronomic and economic value spanning stress resistance, quality traits, growth development, and yield regulation (Table 2).

**Table 2:** Application of GWAS in horticultural crops for trait dissection.

**Crop**	**Population size**	**Traits**	**Number of associate SNP/QTL/candidate genes**	**Key candidate gene(s)**	**Reference**
Chinese chestnut	151 accessions	Total bur weight (TBW), maximum total bur weight (MTBW), leaf length (LL), leaf width (LW), leaf length to width ratio (LLWR), single nut weight (SNW)	82 SNPs, 45 loci, 6 candidate genes	CmAP2* (SNW); CmCIB1* (LL)	[53]
Spinach	305 accessions	Bolting, flowering, oxalate content, sex expression, downy mildew (DM) resistance, DM incidence, plant morphology, leaf traits, petiole traits, etc.	372 significant signals (12 traits) and 34 signals (8 traits)	*SOV3g001250* (DM resistance); *SOV1g011880* (organ size); *SOV6g040410* (leaf texture)	[54]
Strawberry	319 accessions	Winter survival, plant vigor, flowering time, runnering vigor, male fertility, female fertility, berry appearance, productivity, etc.	39 QTLs (11 consistent, 28 putative)	*ADH* and *SWEET3* (winter survival); *TFL1* (flowering time)	[23]
Strawberry	305 accessions	97 volatiles (esters, terpenes, aldehydes, alcohols, acids, ketones, lactones, etc.)	62 significant signals	FaASa1* (methyl anthranilate); FaOMT* (mesifurane); *FaNES1* (terpenes)	[55]
Tea plant	92 accessions	Cold tolerance	10 significant associated SNPs	CsUGT71A60* (cold tolerance)	[56]
Tea plant	155 accessions	Leaf color, timing of bud flush (TBF), catechin content, terpene content, etc.	887 986 SVs; 35 significant SNPs (for bud flush timing)	*DREB2A-like* (TBF); *CsDIOX* (catechin); *CsAFS1* (α-farnesene)	[26]
Tomato	317 accessions	Cold tolerance (evaluated by electrolyte leakage and tolerance score)	SNPs associated with a 27-bp InDel in SlBBX31 promoter	SIBBX31* (cold tolerance)	2
Tomato	398 accessions	Flavor-associated chemicals (sugars, acids, volatiles)	251 association signals for 20 traits	Lin5* (sugar content); E8* (guaiacol and methylsalicylate)	[57]
Sweet potato	104 accessions	Gene expression variation in storage roots, anthocyanin biosynthesis, etc.	724 438 high-confidence SNPs; 4408 eQTLs (2261 local eQTLs, 2147 distant eQTLs)	*IbMYB1–2* (eQTL; root flesh color)	[58]
Sweet potato	294 accessions	23 agronomic traits (e.g. tuberous root weight, appearance, flesh color, high-density planting traits)	6 828 068 SNPs; 650 unique QTLs (353 associated with BLUP phenotypic values)	IbEXPA4* (root weight); *IbOr* (flesh color)	[59]
Cauliflower	691 accessions	Curd biogenesis, stem height (SH), curd diameter (CD), etc. (7 traits)	9 dominant association signals	BOB06G135460* (SH)	[60]
Persian walnut	285 accessions	Shell thickness	347 significant SNPs; 19 significant SVs	JrUGP*, JrMYB308* (shell thickness)	[61]
Chrysanthemum	137 accessions	Four active compounds (total flavonoids, chlorogenic acid, luteolin, isochlorogenic acid A)	59 significant SNPs	*QUA1* (luteolin)	[62]
Cucumber	115 accessions	SVs (including gynoecy, tuberculate fruit trait)	26 788 SVs; 1 significant CNV (for gynoecy)	*Csa5G577350* (tuberculate fruit trait); *ACS1/Csa6G496450* (gynoecy)	[25]
Cucumber	220 accessions	Salt tolerance (seedling stage, evaluated by salt injury index)	7 repeatedly detected loci	*CsaV3_2G035120*, *CsaV3_3G023710*, *CsaV3_4G033150*, *CsaV3_5G023530*, *CsaV3_6G009810* (salt tolerance)	[63]
Potato	108 accessions	25 agronomic traits (e.g. tuber size, small-sized tuber weight, tuber thickness)	50 candidate loci associated with 15 traits	GA20ox1, PHL (tuber aspect ratio); *OSM34*, *SAUR66*, *SAUR20*, *SWEET7* (small-sized tuber weight); *RGL2*, *GA2ox6*, *PHYB* (tuber thickness)	[64]
Potato	248 accessions	1258 metabolites (flavonoids, phenolic acids, phospholipids, etc.)	9321 presence–absence variations (PAVs)	SSIII/DM8C02G20430* (starch); *DM8C01G46870* (phenolic acids); *DM8C10G16540* (amino acids)	[65]
Pepper	148 accessions	4 fruit traits (fruit color, fruit width, fruit length, fruit weight)	12 significant QTLs	*CCS/Capana06g000615* (fruit color)	[51]
Sand pear	312 accessions	11 agronomic traits (8 fruit quality traits, 3 phenological traits)	37 loci (fruit quality); 5 loci (phenological traits)	PbrSTONE/Pbr005042* (stone cell content)	[50]
Apple	204 accessions	Fruit ripening	3 variations (2 SNPs and 1 58-bp InDel)	*MdNAC18.1/MD03G122260*0* (fruit ripening)	[66]
Apple	270 accessions	Metabolites (tannins, organic acids, phenolic acids, flavonoids, lipids, etc.), fruit weight, firmness	222 877 significant SNPs associated with 2205 metabolites	Myb9-like*, LAR* (tannins); FAD2* (LPE 18:1); NAP* (SA); ABCG25* (ABA)	[49]
Tree peony	271 accessions	Floral organ number (petal, stamen, carpel)	255 significant SNP-trait associations; 86 GWAS-related cis-eQTLs; 3188 trans-eQTL gene pairs	*ASIL2* (petal number); *REV* (carpel number); *KNAT3* (stamen number)	[67]
Lettuce	381 accessions	Flowering time, leaf color, leaf shape (curvature, lobing), leaf length, leaf width, etc.	51 for flowering time; 11 for leaf color; 5 for leaf shape; multiple loci for leaf size	LsphyB*, LsphyC* (flowering time); bHLH*, ANS* (leaf color and stem color); LsTCP4* (leaf shape); ZFP* (lobed leaves)	[17]
Peach	129 accessions	12 agronomic traits (e.g. fruit shape, nonacidity, fruit hairiness, flesh adhesion, fruit weight, soluble solid content)	Multiple loci (e.g. 1 for fruit shape, 1 for nonacidity, 33 for fruit weight, 24 for soluble solid content)	*PpCAD1/ppa003772m* (fruit shape); *ppa006339m* (nonacidity fruit)	[68]
Grapevine	466 accessions	29 agronomic traits (bunch traits, fruit contents, berry traits, fruit size, skin traits)	148 QTLs (50.7% newly identified); 8 591 818 SNPs, 513 969 InDels, 236 449 SVs	*Vitvi011427* (berry length); *Vitvi030206* (number of seeds); *RHM1* (sucrose content)	[69]
Sesame	366 germplasm lines	Seed coat color (evaluated by L, a, b color space values, BLUP values, and principal components)	224 SNPs (for color space values); 119 SNPs (for BLUP values); 197 SNPs (for principal components); 35 SNPs detected in >6 environments	*SIN_1016759*, *SIN_1023237*, *SIN_1006022*, *SIN_1023226*, *SIN_1024895* (seed coat color)	[22]

Genes marked with an asterisk have been functionally validated through experimental approaches.

### Genetic dissection of stress resistance traits

Horticultural crops face severe biotic and abiotic stress challenges that directly impact yield and distribution. GWAS demonstrates strong efficacy in dissecting stress resistance, revealing natural variation that enriches genetic resources for breeding. Regarding cold resistance mechanisms, GWAS integrated with proteomics in tea plant (*Camellia sinensis*) identified *CsUGT71A60*, enhancing tea plant cold tolerance via cytokinin homeostasis modulation [56]. Tomato studies revealed promoter variation in *SlBBX31* associated with cold tolerance [2]. For heat stress, combined GWAS and transcriptome analysis in grapevine revealed that the WRKY transcription factor TTC4 enhances thermotolerance by activating *heat shock protein* (*HSP*) and *ascorbate peroxidase* (*APX*) gene expression [48]. Regarding salt stress, GWAS in cucumber (*Cucumis sativus*) detected multiple QTLs related to seedling salt tolerance [63], while tomato GWAS dissected genetic variation in sodium exclusion mechanisms within wild germplasm [70]. Notably, GWAS with map-based cloning in peach identified *PpNLR1*, where a 20-bp InDel regulates aphid resistance via jasmonic acid signaling [71]. Additionally, GWAS has been extensively applied in pepper disease resistance research, where integration with QTL mapping identified multiple key loci conferring resistance to *Phytophthora capsici* [72]. These achievements deepen our understanding of plant stress resistance mechanisms and provide crucial targets for molecular design breeding. The integration of multi-omics technologies will further amplify the role of GWAS in improving stress resistance.

### Polygenic regulatory networks underpinning quality traits

Quality traits in horticultural crops directly influence consumer preference and market value. Systematic analysis of natural variation by GWAS has elucidated the genetic basis of flavor, color, texture, and nutritional traits. In tomato, GWAS of flavor metabolisms identified *Lin5* and *E8* genes regulating sugar and volatile compounds [57]. Tea plant mGWAS identified *UGT* and *CCoAOMT* as regulators of theanine and catechin accumulation [73]. For color research, studies in *Prunus mume* have identified the *R2R3 MYB* gene *MYB108* as a critical locus for petal color determination via GWAS [74]. Regarding fruit texture and nutritional quality, a litchi (*Litchi chinensis* Sonn.) GWAS identified the invertase gene *LcSAI* as a key regulator of sugar composition and sweetness [75]. Peach studies associated *PpCAD1* polymorphisms with flesh texture [68]. Potato pangenome-based GWAS identified PAVs associated with starch content and flavonoid accumulation, including an *SSIII*-downstream insertion boosting starch [65]. Multi-omics integration enhances mechanistic insights in metabolome-based GWAS. In grape, this approach validated the dual function of *VvGGPPS-LSU*, and in tea it helped dissect the flavonoid biosynthesis. These findings provide end-to-end solutions for molecular design breeding targeting quality traits.

### Evolutionary insights into yield and developmental traits

Yield and developmental traits in horticultural crops directly determine economic value, with GWAS serving as a key tool for dissecting their genetic complexity. In fruit development, apple GWAS research revealed that an InDel variant in the *MdNAC18.1* promoter accelerates fruit ripening by regulating ethylene synthesis and cell wall softening genes [66]. Strawberry multimodel GWAS identified QTLs in chromosome 3D controlling shape, size, and yield [23]. In addition, lettuce MAGIC GWAS revealed LsphyB/LsphyC loss-of-function mutations delaying flowering and identified a novel zinc finger gene regulating the development of lobed leaves [17]. Cauliflower (*Brassica oleracea* L. var. *botrytis*) GWAS detected a nonsynonymous SNP and a 3-bp deletion in exon 6 of the zinc finger protein gene Bo6G135460 positively regulating stem height. Functional validation using CRISPR-Cas9 knockout of this gene resulted in significantly shorter stems, while overexpression lines displayed elongated stems, confirming its causal role. This provides a clear example of a GWAS-identified locus being rapidly translated into a functional genetic resource for breeding [60]. Mulberry (*Morus* spp.) WGS-GWAS elucidated domestication-related loci for flowering time, leaf size, and sex determination [24]. In yield trait dissection, tomato GWAS revealed pleiotropic loci synchronously regulating flowering time, fruit size, and yield [15]. In pomegranate (*Punica granatum* L.), GWAS has identified a locus on chromosome 6 significantly associated with 100-grain weight. A cytochrome P450 87A3-like gene within this region is proposed to regulate grain size through the auxin response pathway, thereby affecting the internal structure of the fruit [76]. By continuously integrating multi-omics data and refining analytical methods, GWAS will play an increasingly vital role in breeding new cultivars with high yield, superior quality, and enhanced stress resistance.

Collectively, these studies demonstrate the power of GWAS in dissecting the genetic basis of stress resistance, quality formation, and yield-related traits in horticultural crops. However, despite these advances, most trait-associated loci have been identified primarily through SNP-based analyses anchored to a single linear reference genome. This approach inherently constrains the resolution of causal variant discovery, particularly for SVs and presence–absence variations that are widespread in horticultural genomes. As genetic architectures become increasingly complex, analytical frameworks capable of capturing multivariant and multilayer regulatory features are urgently required. Graph pangenomes provide such a framework by representing genomic sequences from multiple individuals within a unified graph-based reference. When combined with multi-omics integration, this strategy offers a powerful paradigm for precise variant resolution and mechanistic interpretation.

## Graph pangenome and multi-omics integration empower precise gene fine mapping

### Multimarker colocalization empowers gene fine-mapping: graph pangenome technology enables variant-integrated analysis

Within a graph pangenome-enabled framework, GWAS can move beyond single-variant testing to systematically integrate multiple classes of genetic variation into fine-mapping analyses. By jointly modeling SNPs, SVs, and PAVs, graph-based association strategies enable multimarker colocalization and substantially improve the resolution of causal variant identification for complex horticultural traits. For instance, Li *et al.* [77] constructed a peach pangenome and panvariome representing 1020 accessions, cataloging >10.5 million variants including SNPs, InDels, SVs, CNVs, translocation/inversion polymorphisms, and PAVs. They subsequently developed GWASPV, a unified analytical method integrating multiple variant types in a single step. GWASPV successfully identified causal genes and variants for traits such as fruit shape and flesh color, outperforming conventional GWAS in statistical power. This superior performance demonstrates the efficacy of multivariant colocalization analysis for dissecting complex traits. Similarly, the tomato super pangenome integrated genomes from nine wild and two cultivated species while incorporating 360 189 SVs. Graph-based SV-GWAS revealed trait associations at 21.3% of loci undetected by SNP-GWAS, underscoring the necessity of variant integration for comprehensive analysis [27]. Building on this precision, a graph-based tea pangenome that integrated variations from 22 cultivars was harnessed. A combined analysis of SNP and SV markers then narrowed the QTL interval for bud flush timing (TBF) from 38.45 Mb down to a remarkably focused 607-kb region, which contained only 25 candidate genes. Subsequent SV-GWAS then precisely identified functional allelic variation within the key regulatory gene *DREB2A-like,* achieving high-confidence localization [26]. These examples highlight how graph pangenomes dramatically enhance the breadth and resolution of genetic variant detection and association mapping.

### Deciphering functional mechanisms: multi-omics integration illuminates the path

While graph pangenomes expand the genomic landscape, multi-omics integration provides the crucial depth needed to elucidate the functional pathways connecting genetic variation to complex phenotypes. Rapid advances in omics technologies have transformed GWAS from a genome-to-phenotype approach to a systemic strategy integrating transcriptomics, metabolomics, phenomics, and epigenomics, effectively overcoming single-layer mapping limitations [78]. Transcriptome integration is pivotal, elucidating the direct regulatory impact of gene expression regulation on phenotypes [79]. Peng *et al.* [67] integrated GWAS with transcriptomics in tree peony to dissect genetic bases and coexpression networks underlying floral organ number variation. In sweet potato (*Ipomoea batatas*), joint transcriptome-GWAS analysis identified 4408 eQTLs regulating 3646 genes in storage roots. An eQTL hotspot on chromosome 12 harbors *IbMYB1–2*, which regulates flesh color by activating anthocyanin biosynthesis. This finding demonstrates a direct mechanistic link between transcriptional networks and phenotypic traits [58]. In rapeseed (*Brassica napus*), GWAS with spatiotemporal transcriptomics validated that BnaA02.SE affects yield by regulating cell proliferation-related gene expression [80]. mGWAS integration dissects the genetic architecture of biochemical pathways. In peach fruit metabolite research, Cao *et al.* combined mGWAS with eQTL mapping to identify genomic hotspots regulating metabolites, including flavonoids and citric acid. These hotspots exhibit artificial selection signatures during domestication and associate with functional traits including antioxidant and anticancer activities [81]. Similarly, a comprehensive mGWAS in the tea plant integrated pantranscriptomics, metabolomics, and multi-omics analyses to decode regulatory networks for 212 flavor metabolites. It identified 3843 candidate genes and 3407 eQTLs, pinpointing key regulators CsANS (proanthocyanidin variation) and F3′5′H (catechin distribution divergence), providing crucial insights for tea flavor improvement breeding [82]. Integrated multi-omics QTL mapping analysis of tomato fruit quality and disease resistance elucidated the steroidal glycoalkaloid transformation pathway governed by *GAME31/GAME5*. Metabolome-GWAS association networks further identified antifungal metabolites, including pantothenic acid. This revealed a synergistic genetic network in which wild introgression drives the coordinated evolutionary remodeling of both defense metabolism and flavor formation during fruit ripening [83].

Integrating GWAS with epigenomics further expands the research dimension. In tomato, the integration of mGWAS with methylome-wide association studies (mEWAS) constructed regulatory networks that link metabolism, genetic variation, and DNA methylation. This work demonstrated that DNA methylation variants drove the evolution of metabolic diversity during domestication [84]. Walnut (*Juglans regia*) shell development research combined GWAS with methylomics to elucidate DNA methylation regulation of shell development genes [61]. True systems-level insights emerge from the integration of multiple omics layers. For example, Dominic Knoch *et al.* [85] integrated GWAS with transcriptome, proteome, and metabolome data in rapeseed to dissect cascade regulation from genetic loci to metabolic pathways, identifying key regulatory genes during biomass accumulation. By contrast, in horticultural crops, proteome-level integration—particularly proteome-wide association analysis (PWAS)—remains relatively underexplored, with only a few representative examples reported to date. In multi-omics studies of pepper fruit traits, Liu *et al.* integrated TWAS and PWAS within a recombinant inbred line (RIL) population, locating 12 core QTLs for fruit color, size, and weight. These analyses further revealed cascade effects where genomic hotspots like LG6–1 simultaneously regulate gene expression (eQTL) and protein abundance (pQTL) [51]. Beyond proteome-level association, fine-mapping causal variants underlying horticultural traits increasingly relies on the integration of diverse genomic layers. To pinpoint causal variation, Fan *et al.* integrated multiple genomic approaches, including haplotype-phased genomes, structural variant analysis, eQTL mapping, and volatile mGWAS. Specifically, they identified a 28-bp exon deletion in the FaOMT gene as the functional variant responsible for methylfuranone synthesis. This frameshift mutation illustrates how structural variants can drive metabolic diversity by modulating gene expression [55].

While these multi-omics approaches significantly enhance the identification of candidate causal variants and mechanistic hypotheses, establishing definitive causality from association signals remains a central challenge. Critically, establishing causality requires integrating multiple omics layers to triangulate evidence. For instance, colocalization analysis between eQTL and mGWAS can be employed. This approach tests whether the same genetic variant influences both gene expression levels and metabolite abundance. If a shared variant is found, it provides strong evidence for a causal cascade: the genetic variant regulates gene expression (eQTL), and the altered expression of that gene subsequently affects the accumulation of a specific metabolite (mGWAS signal). This establishes a mechanistic link within the molecular pathway. These molecular pathways often underpin complex horticultural traits such as flavor, color, and stress resilience. Therefore, linking genetic variants to intermediate molecular phenotypes enables stronger causal hypotheses for how genomic variation shapes complex traits. Mendelian randomization (MR) analysis, which uses genetic variants as instrumental variables to infer causal relationships between an exposure (e.g. gene expression) and an outcome (e.g. disease phenotype), provides a powerful statistical framework for causal inference. When integrated with multi-omics data, MR helps distinguish whether observed changes in intermediate molecular phenotypes are genuine causal drivers of the trait or merely consequences of confounding factors [86]. Consequently, the integration of MR into multi-omics frameworks offers a robust analytical tool for advancing from correlative associations to causal mechanisms, thereby significantly strengthening the reliability of candidate gene prioritization and functional validation strategies. This integrated approach is especially valuable for elucidating complex horticultural traits governed by intricate gene regulatory and metabolic networks.

### Synergy toward precision

**Figure 2:**
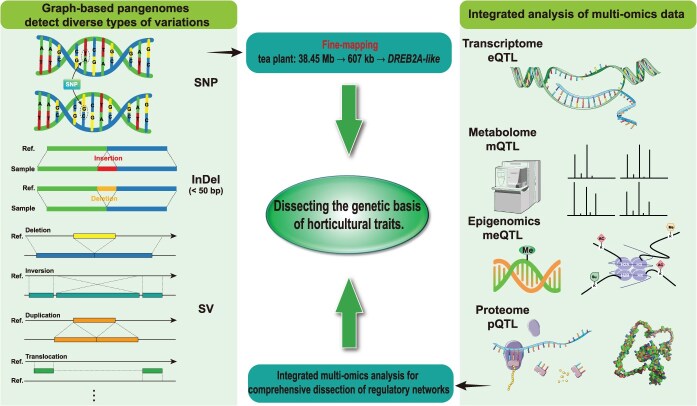
Graph pangenome–multi-omics synergy system: precise gene dissection from structural variants to phenotypes.

Collectively, the synergistic application of graph pangenome frameworks and multi-omics integration enables the systematic dissection of regulatory cascades from genomic variation to phenotypic manifestation. Graph pangenomes provide the comprehensive variant catalog, particularly capturing elusive SVs and PAVs, while multi-omics layers (transcriptome, proteome, metabolome, epigenome) illuminate the functional consequences and interactions across the biological hierarchy (Fig. 2). Figure 2 schematically illustrates this integrated synergy system, where graph pangenomes supply a comprehensive variant map, and multi-omics layers decode the functional cascade from genomic variation to phenotypic traits, thereby enabling precise gene dissection. This combined approach is essential for pinpointing key causal loci and elucidating the multidimensional ‘variant → gene expression → protein function → metabolic pathway → complex trait’ interplay. With the advent of spatial omics and single-cell technologies, future research will further unveil the dynamic spatiotemporal regulatory blueprints of horticultural traits. This progress will build upon the foundation of precise gene localization established by graph pangenomes and integrated multi-omics. Looking forward, the integration of single-cell RNA sequencing (scRNA-seq) with GWAS promises to enable the dissection of cell-type-specific genetic effects, potentially elucidating how genetic variants modulate gene expression across distinct cell populations. This approach could thus be pivotal for deciphering the complexity of tissues such as fruits or floral organs. Similarly, coupling spatial transcriptomics with GWAS holds the potential to map gene expression onto tissue architecture and uncover spatially patterned genetic regulation. This would be critical for dissecting the genetic basis of traits such as fruit development, pigment distribution, and meristem activity [87].

## From gene discovery to precision design: progress and future prospects of GWAS in horticultural crops

GWAS has emerged as a core tool for dissecting the genetic basis of complex traits in horticultural crops, driving a paradigm shift from gene discovery to design breeding. By deeply mining natural genetic variation, GWAS has achieved breakthroughs in key traits like stress resistance, quality formation, and yield regulation, with numerous critical loci identified across various species (as summarized in Table 2). These discoveries exemplify the power of GWAS in linking genomic variation to phenotypic diversity. Notably, several GWAS-identified loci have already been translated into practical breeding tools. A prime example is the discovery of *Sl-ALMT9*, a key gene regulating fruit malate content and aluminum tolerance in tomato. Through integrated GWAS and BSA-seq, a 3-bp InDel in its promoter was found to enhance malate accumulation by disrupting a W-box. This InDel was subsequently developed into a cleaved amplified polymorphic sequence (CAPS) marker, enabling efficient genotyping and marker-assisted selection for high malate content in commercial tomato hybrids [52]. Continuous optimization of the GWAS workflow has significantly enhanced its resolving power. As summarized in previous sections, advances in population design, high-throughput phenomics, graph-pangenome frameworks, multi-omics integration, and statistical modeling have collectively enhanced the resolution and biological interpretability of GWAS outputs. These studies not only pinpoint key genetic loci but also reveal the cascade regulatory networks linking ‘variant → gene expression → metabolic pathway → phenotype’.

Despite these remarkable advances, several inherent limitations of GWAS must be acknowledged to contextualize its applications and guide future improvements within horticulture. A significant challenge is the detection of rare variants (MAF <0.01), as they typically yield low statistical power in standard diversity panels. Moreover, highly polygenic traits, governed by many loci with small effect sizes, remain challenging to dissect comprehensively; current models often fail to capture these subtle contributions against background noise. A further persistent challenge involves the accurate incorporation of G × E interactions, which are particularly influential in perennial horticultural species cultivated across diverse agroecological zones. Even when employing multi-environment trials and advanced models, disentangling G × E effects from genetic signals demands large, well-replicated phenotypic datasets, whose collection is resource intensive. Other confounding factors include population stratification, reference genome bias, and incomplete variant detection in structurally complex genomes (such as polyploids), all of which can generate false associations or mask causal loci. Overcoming these limitations will require sustained innovation in population design, phenotyping platforms, statistical methodologies, and the integration of pangenomic resources to render GWAS-derived insights both biologically insightful and practically actionable for breeding.

**Figure 3:**
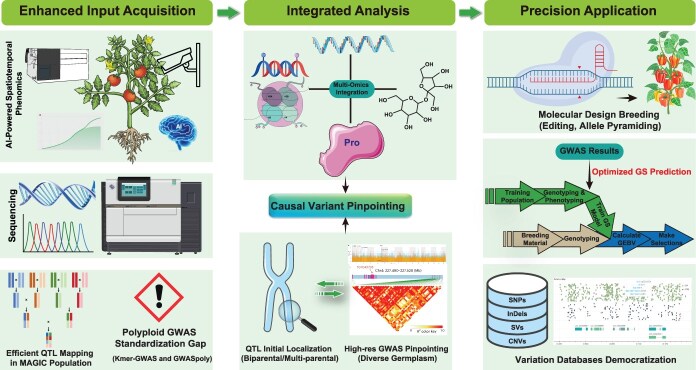
Synergistic pipeline for advancing GWAS in horticultural crop improvement. The LD analysis plot is reproduced from Chen et al. [26].

The continued evolution and impact of GWAS in horticultural crop improvement hinge on synergistic advancements across a cohesive pipeline encompassing enhanced input acquisition, integrated analysis, and precision application (Fig. 3). Figure 3 illustrates this cohesive framework, outlining the key stages from enriched genetic and phenotypic input, through causal inference via integrated multi-omics and QTL analysis, to the translation of discoveries into breeding applications for cultivar development.

Revolutionizing the foundation, enhanced input acquisition focuses on capturing richer genetic and phenotypic data. Artificial intelligence (AI) will catalyze a paradigm shift in phenomics, enabling automated, high-throughput, and spatiotemporally continuous measurement. This capability is critical for dissecting complex, holistic traits, such as dynamic stress responses or intricate fruit development pathways, into quantifiable subphenotypes. Consequently, it can uncover previously obscured major-effect genes and nuanced gene–environment interactions. This approach effectively overcomes the limitations of labor-intensive manual scoring [88, 89]. Concurrently, genotyping advancements will utilize long-read sequencing for comprehensive structural variant detection. However, a persistent frontier that demands significant innovation is the development of robust, standardized, and widely applicable GWAS models tailored for polyploid species. While promising solutions like kmer-GWAS and GWASpoly exist, the field lacks universal adoption and consensus frameworks. Complementing these technological strides, strategic population design at the source remains paramount. MAGIC populations and similarly structured designs are meticulously constructed to minimize confounding population structure while enriching target allelic diversity. These populations have proven exceptionally powerful for initial QTL detection and serve as indispensable resources for subsequent fine-mapping efforts.

Building upon this enriched data foundation, integrated multi-omics analysis will unlock unprecedented causal inference and mechanistic understanding. The future power of GWAS will be amplified by its deep convergence with multiple omics layers, such as transcriptomics, epigenomics, proteomics, and metabolomics. This integration maps the intricate spatiotemporal dynamics of gene regulation, protein function, and metabolic flux. Equally crucial is the strategic synergy achieved by combining GWAS with traditional QTL mapping. This powerful combination harnesses complementary strengths. First, QTL analysis in controlled biparental or multiparental populations provides robust initial localization of trait-associated genomic intervals, with reduced confounding. Subsequently, high-resolution GWAS conducted within these targeted intervals across diverse germplasm enables precise pinpointing of causal polymorphisms and candidate genes. This precision is further enhanced by integrating functional omics evidence. This sequential strategy, as exemplified by the identification of key bud dormancy genes in tea plant, bridges association signals to causal mechanisms and accelerates functional validation. Ultimately, this integrated paradigm harnesses the breadth of natural diversity association, precision of linkage mapping, and depth of multi-omics to deconvolute the complete cascade from genomic variation through molecular intermediates (gene expression, protein function, metabolic pathway) to the final horticultural phenotype.

The ultimate goal lies in precision application, translating genetic discoveries into tangible breeding gains. GWAS-derived causal variants and regulatory insights will directly fuel molecular design breeding, enabling targeted genome editing and sophisticated allele pyramiding. Critically, GWAS results will be harnessed to enhance the accuracy and efficiency of genomic selection (GS) models; incorporating trait-associated SNPs as fixed effects or using GWAS-derived weights significantly improves breeding value prediction, shortening breeding cycles [90]. Furthermore, a pivotal advancement will be the democratization of genomic resources. This can be achieved by establishing and widely adopting centralized, integrated variation databases alongside accessible analytical platforms. A prime example is RiceVarMap, a comprehensive database for rice genomic variation and its functional annotation [91]. These platforms unify diverse resources, including pangenomes, variant annotations, GWAS results, multi-omics data, and functional validation information, within intuitive interfaces. They serve as essential conduits that empower breeders and researchers, even those without specialized bioinformatics expertise, to translate discoveries into actionable breeding decisions. In doing so, they bridge the critical gap between computational genetics and applied breeding.

Collectively, these synergistic advancements across the GWAS pipeline—from enriched data input through integrated causal analysis to precision breeding application—will fundamentally transform horticultural improvement, enabling the predictive design of resilient, high-performing cultivars to meet the challenges of a changing world.

The integrative framework and future roadmap presented above underscore the central contribution of this review: technology synergy and breeding translation. This review not only synthesizes recent advances in GWAS applications for horticultural crops but also establishes a systematic framework that emphasizes technology synergy and breeding translation as its core contribution. Distinct from earlier reviews, we provide an in-depth discussion on the integration of graph pangenomes with multi-omics approaches, including emerging spatial omics, and delineate clear, actionable pathways for implementing GWAS discoveries into precision breeding programs. By highlighting the synergistic convergence of high-resolution genomics, AI-enhanced phenomics, advanced statistical models, and functional validation strategies, this work offers a forward-looking perspective that bridges cutting-edge genomic research with practical horticultural improvement. Our integrated approach not only captures the state-of-the-art but also charts a roadmap for translating genetic insights into resilient, high-performing cultivars, thereby underscoring the unique value of this review in advancing both theory and practice in horticultural genomics and breeding.
